# Solitary colonic metastasis from renal cell carcinoma presenting as a surgical emergency nine years post-nephrectomy

**DOI:** 10.1186/1477-7819-8-54

**Published:** 2010-06-29

**Authors:** Alka M Jadav, Sri G Thrumurthy, Bernard A DeSousa

**Affiliations:** 1 Department of Lower Gastrointestinal Surgery, Royal Preston Hospital, Preston, PR2 9HT, UK; 2 University Surgical Unit, National Hospital of Sri Lanka, Colombo 10, Sri Lanka; 3 Department of Lower Gastrointestinal Surgery, Fairfield General Hospital, Manchester, BL9 7TD, UK

## Abstract

Late colonic metastasis following curative surgery for renal cell carcinoma has rarely been described. We present the first reported case of solitary colonic renal cell carcinoma metastasis presenting as an intra-abdominal bleed, nine years post-nephrectomy.

## Background

The worldwide incidence of renal cell carcinoma (RCC) is approximately 209 000 new cases per year with a mortality of 102 000 deaths per year. This accounts for 3% of all adult malignancies. Metastatic disease may be present in up to 25% of patients at the time of diagnosis [1,2].

Intestinal metastasis from RCC is uncommon. The commonest site of distant metastasis in 1451 autopsy cases with RCC was in the lungs (76%), followed by lymph nodes, bones and liver [3]. RCC very rarely metastasizes to the colon - a comprehensive Medline search revealed only 7 reported cases to date, of post-nephrectomy colonic metastasis from RCC [4-10]. This case represents the first incidence of late colonic RCC metastasis presenting as a surgical emergency in the way of an intra-abdominal bleed.

## Case Presentation

A 65-year-old woman presented to casualty with acute abdominal pain and collapse. The only significant history was of a left nephrectomy for clear cell renal carcinoma nine years previously, from which she had made a full recovery, recently being discharged from further follow-up. The patient recalled that her RCC had been excised with tumour-free margins - no further information was available.

Examination revealed generalised abdominal tenderness with a normal haemoglobin of 11.4 g/dL. Portable ultrasound scan excluded an abdominal aortic aneurysm. A few hours later, she became haemodynamically unstable with marked abdominal distension. Repeat bloods showed a drop in haemoglobin to 7.7 g/dL. There had been no sign of haematemesis, melaena or fresh rectal bleeding. At emergency laparotomy, an actively bleeding mass was found attached to the surface of the mid-transverse colon. This was excised locally with the resulting colonic defect closed in 2 layers. No other lesions were noted within the abdominal cavity.

Macroscopic examination revealed a 6 × 6 cm soft brown tumour with central necrosis. Histology of the lesion demonstrated a clear cell tumour - a metastasis from the original renal cell carcinoma removed nine years previously. Subsequent computed tomography (CT) of the thorax and abdomen excluded any further metastatic disease. As such, a conservative approach without immunotherapy was adopted and the patient was followed-up with regular clinical examination and CT scans. No evidence of further recurrence has been demonstrated six years following her laparotomy.

## Conclusions

Uchida et al have stated that if patients with RCC undergo curative nephrectomy and subsequently develop recurrence, this usually occurs within five years post-operatively (i.e. early recurrence) [8]. Out of 239 patients who had no distant metastasis at the time of initial diagnosis, 68 patients had recurrence after nephrectomy. 84% of these were within the first five years following surgery. Late recurrence of RCC occurs in as many as 11% of patients surviving ten years or more, and the longest reported interval from nephrectomy to recurrence is 31 years [7,9,10].

The biological behaviour of RCC is variable, and the prognosis unpredictable. Despite it being a male-predominant disease (2:1), the predominance of women among patients with late recurrence and their better survival rate may suggest an endocrine influence on the activity of the tumour [1,4,6,9,11]. Late recurrence is not only more likely to occur in women but also in individuals with well-differentiated tumours [6,11]. This supports the importance of prognostic markers like the Fuhrman nuclear grade and tumour-node-metastasis (TNM) staging in determining future metastatic potential of RCC [1,12]. Surgical treatment has been reported to improve survival after late recurrence in patients with solitary metastasis that is confined to one organ. The surgical approach thus remains the most therapeutic option whenever delayed recurrence is resectable [12,13].

In summary, recurrence of RCC more than five years after nephrectomy is not a rare event, and is one of the particular characteristics of RCC [14]. However, delayed recurrence cannot be predicted at the time of treatment of the primary lesion [15]. Therefore, careful long-term follow-up may be beneficial for patients with a history of RCC even after undergoing a curative nephrectomy [6,9]. If patients with a history of previous RCC present with an abdominal complaint, surgeons should always consider potential recurrences and seek to exclude further metastases.

## Consent

Written informed consent was obtained from the patient for publication of this case report and any accompanying images. A copy of the written consent is available for review by the Editor-in-Chief of this journal.

## Authors' contributions

AMJ and BAD were responsible for delivering patient care. AMJ and SGT contributed equally towards to drafting of the manuscript while BAD provided overall supervision and edited the final version of the manuscript. All authors read and approved the final manuscript.

## References

[B1] Rini BI Campbell SC Escudier B Renal cell carcinoma Lancet 2009 373 9669 1119 32 10.1016/S0140-6736(09)60229-4 19269025

[B2] McNichols DW Segura JW Renal cell carcinoma- long term survival and late recurrence J Urol 1981 126 17 23 7253072 10.1016/s0022-5347(17)54359-1

[B3] Saitoh H Distant metastasis of renal adenocarcinoma Cancer 1981 48 1487 1491 10.1002/1097-0142(19810915)48:6<1487::AID-CNCR2820480635>3.0.CO;2-9 7272969

[B4] Thomason PA Peterson LS Staniunas RJ Solitary colonic metastasis from renal-cell carcinoma 17 years after nephrectomy. Report of a case Dis Colon Rectum 1991 34 8 709 12 10.1007/BF02050356 1855429

[B5] Diaz-Candamio MJ Pombo S Pombo F Colonic metastasis from renal cell carcinoma: helical-CT demonstration Eur Radiol 2000 10 1 139 40 10.1007/s003300050020 10663731

[B6] Utsunomiya K Yamamoto H Koiwai H Kirii Y Kashiwagi H Konishi F Kurihara K Kawai T Sugano K Solitary colonic metastasis from renal cell carcinoma 9 years after nephrectomy: report of a case Int J Colorectal Dis 2001 16 3 193 4 10.1007/s003840000281 11459294

[B7] Tokonabe S Sugimoto M Komine Y Horii H Matsukuma S Solitary colonic metastasis of renal cell carcinoma seven years after nephrectomy: a case report Int J Urol 1996 3 6 501 3 10.1111/j.1442-2042.1996.tb00585.x 9170582

[B8] Uchida K Miyao N Masumori N Takahashi A Oda T Yanase M Kitamura H Itoh N Sato M Tsukamoto T Recurrence of renal cell carcinoma more than 5 years after nephrectomy Int J Urol 2002 9 1 19 23 10.1046/j.1442-2042.2002.00418.x 11972645

[B9] Yetkin G Uludag M Ozagari A Solitary colonic metastasis of renal cell carcinoma Acta Chirurgica Belgica 2008 108 2 264 5 18557158 10.1080/00015458.2008.11680218

[B10] Kradjian RM Bennington JL Renal cell carcinoma recurrence 31 years after nephrectomy Arch Surg 1965 90 192 5 14232942 10.1001/archsurg.1965.01320080016004

[B11] Nakano E Fujioka H Matsuda M Osafune M Takaha M Sonoda T Late recurrence of renal cell carcinoma after nephrectomy Eur Urol 1984 10 5 347 9 6519139 10.1159/000463826

[B12] Cohen HT McGovern FJ Renal-cell carcinoma N Engl J Med 2005 353 23 2477 90 10.1056/NEJMra043172 16339096

[B13] Newmark JR Newmark GM Epstein JI Marshall FF Solitary late recurrence of renal cell carcinoma Urology 1994 43 5 725 8 10.1016/0090-4295(94)90199-6 8165776

[B14] Jain V Shergill GS Gupta K Bhandari RK Case report: Renal cell carcinoma: Unusual metastases Indian J Radiol Imaging 2000 10 249 51

[B15] Tanis PJ van der Gaag NA Busch OR van Gulik TM Gouma DJ Systematic review of pancreatic surgery for metastatic renal cell carcinoma Br J Surg 2009 96 6 579 92 10.1002/bjs.6606 19434703

